# RETRACTED: Alam et al. Empirical Study of Autism Spectrum Disorder Diagnosis Using Facial Images by Improved Transfer Learning Approach. *Bioengineering* 2022, *9*, 710

**DOI:** 10.3390/bioengineering13080894

**Published:** 2026-08-03

**Authors:** Md Shafiul Alam, Muhammad Mahbubur Rashid, Rupal Roy, Ahmed Rimaz Faizabadi, Kishor Datta Gupta, Md Manjurul Ahsan

**Affiliations:** 1 Department of Mechatronics Engineering, International Islamic University Malaysia, Kula Lumpur 43200, Malaysia; alam.s@live.iium.edu.my (M.S.A.); mahbub@iium.edu.my (M.M.R.); rupal.roy@live.iium.edu.my (R.R.); ahmed.rimaz@live.iium.edu.my (A.R.F.); 2 Computer and Information Science, Clark Atlanta University, Atlanta, GA 30314, USA; gupta@cau.edu; 3 School of Industrial and Systems Engineering, University of Oklahoma, Norman, OK 73019, USA

The journal retracts the article titled “Empirical Study of Autism Spectrum Disorder Diagnosis Using Facial Images by Improved Transfer Learning Approach” [1], cited above.

Following publication, concerns were brought to the attention of the Editorial Office regarding the underlying dataset used in this publication [1].

In accordance with the journal’s standard procedures, the Editorial Office and the Editorial Board conducted an investigation that identified significant concerns regarding the study methodology and the reliability of the dataset used. In addition, the investigation was unable to verify that the facial image dataset had originally been collected with appropriate ethical approval or that informed consent had been obtained from the legal guardians of the children whose images were included.

As a result, the Editorial Board has lost confidence in the reliability of the findings and conclusions reported in this article and has decided to retract and remove this paper in accordance with MDPI’s retraction policy.

To maintain the integrity of the scholarly record, the title, author information, and this retraction notice will remain permanently available, while the article itself has been removed. This information has also been transmitted to relevant indexing services.

This retraction was approved by the Editor-in-Chief of the journal *Bioengineering*.

The authors have been informed of this retraction but did not provide a comment on this decision.
